# Novel variants in *CSF1R* associated with adult-onset leukoencephalopathy with axonal spheroids and pigmented glia (ALSP)

**DOI:** 10.1007/s00415-024-12557-0

**Published:** 2024-07-20

**Authors:** Anne S. Schmitz, Janani Raju, Wolfgang Köhler, Stephan Klebe, Khaled Cheheb, Franziska Reschke, Saskia Biskup, Tobias B. Haack, Benjamin Roeben, Melanie Kellner, Nils Rahner, Thomas Bloch, Johannes Lemke, Benjamin Bender, Ludger Schöls, Holger Hengel, Stefanie N. Hayer

**Affiliations:** 1 Hertie Institute for Clinical Brain Research, Tübingen, Germany; 2 German Center for Neurodegenerative Diseases (DZNE), Tübingen, Germany; 3 Department of Neurology, University Hospital Tübingen, Tübingen, Germany; 4 Department of Neurology, University Hospital Leipzig, Leipzig, Germany; 5 Department of Neurology, University Hospital Essen, Essen, Germany; 6 Department of Neurology, DRK Kamillus Klinik, Asbach, Germany; 7 Institute of Human Genetics, University of Leipzig Medical Center, Leipzig, Germany; 8 Center for Rare Diseases, University of Leipzig Medical Center, Leipzig, Germany; 9 Humangenetik und Pränatal-Medizin MVZ GmbH, Eurofins, München, Germany; 10 CeGaT GmbH and Zentrum Für Humangenetik, Tübingen, Germany; 11 Institute of Medical Genetics and Applied Genomics, University Hospital Tübingen, Tübingen, Germany; 12 Institut Für Klinische Genetik Und Tumorgenetik Bonn, Bonn, Germany; 13 Radiologie Am Rhein, Bad Honnef, Germany; 14 Department of Neuroradiology, University Hospital Tübingen, Tübingen, Germany; 15 Institute of Medical and Human Genetics, Charité – Universitätsmedizin Berlin, Berlin, Germany

**Keywords:** CSF1R, CSF1R-related leukoencephalopathy, ALSP, Genetic diagnostics

## Abstract

The *CSF1R* gene, located on chromosome 5, encodes a 108 kDa protein and plays a critical role in regulating myeloid cell function. Mutations in *CSF1R* have been identified as a cause of a rare white matter disease called adult-onset leukoencephalopathy with axonal spheroids and pigmented glia (ALSP, also known as CSF1R-related leukoencephalopathy), characterized by progressive neurological dysfunction. This study aimed to broaden the genetic basis of ALSP by identifying novel *CSF1R* variants in patients with characteristic clinical and imaging features of ALSP. Genetic analysis was performed through whole-exome sequencing or panel analysis for leukodystrophy genes. Variant annotation and classification were conducted using computational tools, and the identified variants were categorized following the recommendations of the American College of Medical Genetics and Genomics (ACMG). To assess the evolutionary conservation of the novel variants within the CSF1R protein, amino acid sequences were compared across different species. The study identified six previously unreported *CSF1R* variants (c.2384G>T, c.2133_2919del, c.1837G>A, c.2304C>A, c.2517G>T, c.2642C>T) in seven patients with ALSP, contributing to the expanding knowledge of the genetic diversity underlying this rare disease. The analysis revealed considerable genetic and clinical heterogeneity among these patients. The findings emphasize the need for a comprehensive understanding of the genetic basis of rare diseases like ALSP and underscored the importance of genetic testing, even in cases with no family history of the disease. The study’s contribution to the growing spectrum of ALSP genetics and phenotypes enhances our knowledge of this condition, which can be crucial for both diagnosis and potential future treatments.

## Introduction

The *CSF1R* gene spans approximately 60 kb on chromosome 5 of the human genome, comprises 21 exons, and encodes a 108 kDa protein [1, 2]. CSF1R protein is a class III receptor tyrosine kinase consisting of an extracellular domain, a transmembrane domain and an intracellular ATP binding site as well as a tyrosine kinase domain [2]. It is activated by two ligands, colony-stimulating factor 1 (CSF-1) and interleukin 34 (IL-34) [2, 3]. Upon ligand binding, the receptor homodimerizes, which results in intracellular autophosphorylation and downstream signaling [4]. CSF1R is mainly expressed on myeloid cells, including monocytes, tissue macrophages, dendritic cells, osteoclasts, and microglia. The survival, proliferation, and differentiation of cells belonging to this lineage is maintained by CSF1R signaling [5, 6].

In 2012, mutations in *CSF1R* were identified as one cause for adult-onset leukoencephalopathy with axonal spheroids and pigmented glia (ALSP) [7]. The inheritance of *CSF1R* mutations in ALSP is autosomal-dominant; a negative family history, however, does not exclude ALSP, as frequent de novo mutations occur, and penetrance is incomplete [8, 9]. Most of the *CSF1R* mutations associated with ALSP are missense mutations located in the tyrosine kinase domain of the receptor [7, 10]. To date, 106 *CSF1R* mutations causing ALSP have been identified [9]. In addition to *CSF1R*, the gene *alanyl-transfer RNA synthetase 2* (*AARS2*) is known to cause a similar disease phenotype [11, 12].

In the brain, CSF1R is predominantly expressed on microglia [13–15]. Since *CSF1R* mutations in ALSP can cause microglia dysfunction [16–18], ALSP is classified as primary microgliopathy [19]. Despite the comprehensive knowledge about CSF1R function in physiological conditions, the exact pathomechanism of ALSP remains unknown.

ALSP patients show a severe neurological disease characterized by substantial cognitive and motor dysfunction due to progressive leukoencephalopathy and brain atrophy [9, 10]. Patients commonly present with progressive cognitive symptoms, including memory impairment, executive dysfunction, and aphasia. Motor deficits such as gait disturbances due to spasticity or parkinsonism are frequently observed, and occasionally tremors or ataxia. Neuropsychiatric symptoms, including depression, apathy and personality changes, are also prevalent. Additionally, patients often exhibit signs of frontal lobe dysfunction, such as disinhibition and impaired judgment. In an advanced state of the disease, epileptic seizures may occur [9, 20]. Neuroimaging typically reveals white matter hyperintensities sparing the temporal lobe and brain atrophy; it may also show persistent diffusion restrictions, corpus callosum abnormalities and white matter calcification [20–22]. The mean age at onset is 43 years but ranges from early adulthood to the 8th decade and occurs earlier in female patients compared to male patients [10, 20]. The mean survival after symptom onset is 7 years [9, 10]. At early stages of the disease, hematopoietic stem cell transplantation represents a therapeutic option, after thorough risk–benefit evaluation [23–25]. Beyond this experimental treatment, no specific therapies for ALSP exist to date.

While the identification of neurofilament light chain (NfL) and chitotriosidase as biomarkers for ALSP has facilitated the diagnostic work-up of the disease [26, 27], diagnostic certainty requires genetic testing and the detection of a pathogenic variant in *CSF1R*. By gene panel and whole exome sequencing, respectively, we identified six novel *CSF1R* variants in seven patients presenting with typical clinical and imaging features of ALSP, which we report here.

## Materials and methods

### Participants and data collection

Patients were recruited during routine clinical care at the neurological department of the University Hospital Tübingen, Germany. Information on age, sex, age at symptom onset, and clinical manifestations were collected from all patients, and a detailed neurological examination was performed by a neurologist (Table 1). All individuals underwent cerebral magnetic resonance imaging (MRI) including T2 fluid-attenuated inversion recovery (FLAIR) and diffusion-weighted imaging (DWI) using 3.0 Tesla devices (Fig. 2).

**Table 1:** Demographic and clinical data

Patient	Mutation	Sex	Origin	Age at onset (years)	First symptom(s)	Symptoms during disease course		Family history	Cerebral MRI features
						Neuropsychiatric	Physical		
Patient 1	c.2384G>T; p.Gly795Val heterozygous	f	German	45	Gait disorder	Impulse control disorder Apraxia Apathy Dementia	Action tremor Progressive gait disorder Spasticity Positive Babinski's sign	Unknown	White matter hyperintensities Global atrophy Diffusion restriction
Patient 2	c.2133_2919del heterozygous	f	Turkish	37	Cognitive deficits	Behavioural changes (aggression, social withdrawal) Aphasia Apraxia Dementia	Parkinsonism Urinary incontinence Faecal incontinence	Negative	White matter hyperintensities Global atrophy
Patient 3	c.1837G>A; p.Val613Met heterozygous	m	German	46	Aphasia Bradykinesia Behavioural changes (indifference)	Dementia	Stooped posture Urinary incontinence	Early-onset dementia in maternal grandmother, mother unaffected	Frontal lobe white matter hyperintensities Global atrophy
Patient 4	c.2304C>A, p.Phe768Leu heterozygous	m	Kazakh	40	Gait disorder Fine motor impairment	Depression Anxiety Dementia Apraxia	Progressive gait disorder Parkinsonism Dysarthria Dysphagia	Negative	White matter hyperintensities Global atrophy Diffusion restriction
Patient 5	c.2517G>T, p.Trp839Cys heterozygous	m	German	46	Cognitive deficits	Aphasia Dementia Apraxia Epilepsy	Facial palsy Progressive gait disorder Dysphagia	Incomplete penetrance/late-onset in mother	White matter hyperintensities Global atrophy Corpus callosum atrophy Diffusion restriction
Patient 6	c.1837G>A; p.Val613Met heterozygous	f	German	51	Gait disorder	Cognitive impairment Pathological laughing Aphasia Apraxia	Dysarthria Progressive gait disorder Fine motor impairment Parkinsonism Dystonia Pyramidal signs	Affected mother	White matter hyperintensities Global atrophy Diffusion restriction
Patient 7	c.2642C>T, p.Ala881Val heterozygous	m	German	44	Behavioural changes (aggression) Depression Cognitive deficits	Dementia Apraxia	Urinary incontinence	Late-onset in mother	White matter hyperintensities Global atrophy Diffusion restriction

### Genetic analysis

Sequencing (whole-exome sequencing or panel analysis) was conducted in routine clinical care either at the Institute of Medical Genetics and Applied Genomics Tübingen, Germany (Patients 3 and 6), the CeGaT Institute Tübingen, Germany (Patients 1, 2 and 4), the Institute of Human Genetics Leipzig, Germany (Patient 5) or the *Institut für Klinische Genetik und Tumorgenetik Bonn*, Germany (Patients 7 and 8).

At the Institute of Medical Genetics and Applied Genomics Tübingen, exome sequencing libraries were generated from genomic DNA using Agilent SureSelect XT Human All Exon V5/V7 enrichment kits (Agilent Technologies) or the TruSeq DNA PCR-Free Kit (Illumina), respectively. Sequencing was performed on a HiSeq2500 or NovaSeq 6000 system (Illumina) as paired-end reads. The sequence data were analyzed using the megSAP pipeline (https://github.com/imgag/megSAP) and aligned to the GRCh37 reference genome.

At the CeGaT Institute Tübingen, genomic DNA was enriched using a custom design Agilent SureSelect in solution kit. Sequencing was performed using barcoded libraries on the SOLiD 5500xl platform (Applied Biosystems by Life Technology). The primary data analysis was performed using Lifescope (versions v2.5-r0 and v2.5-r2.5.1). Only variants (single nucleotide variants (SNVs)/small indels) with a minor allele frequency (MAF) of ≤ 1% in coding and flanking intronic regions (± 8 bp) and the untranslated regions (UTRs) were evaluated.

At the Institute of Human Genetics in Leipzig, exome libraries were generated from genomic DNA using the Illumina TruSeq Rapid Exome Library Preparation kit. Sequencing was performed on the Illumina NextSeq System. Coding sequences ± 8 base pairs of flanking intronic regions were covered by ≥ 20 reads/base per sample. Sequence data were analyzed using the Varvis software (Limbus Medical Technologies GmbH).

At the *Institut für Klinische Genetik und Tumorgenetik* Bonn, Next-Generation Sequencing (NGS) was performed using enrichment methods. The evaluation of sequence variants (SNVs, coding regions as well as adjacent splice sites (± 5 bp)) was carried out using certified pipeline technology (Varvis, Limbus Medical Technologies GmbH). The analysis of larger deletions/duplications was performed through quantitative NGS data analysis (Varvia, Limbus Medical Technologies GmbH). Classification and interpretation of sequence variants were performed using QIAGEN Clinical Insight-Interpret Software (QCI).

### Variant rating and classification

**Fig. 1:**
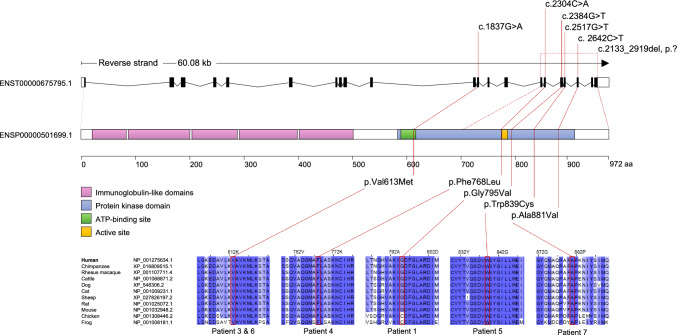
Genomic organization and protein domain structure of CSF1R with characteristics of novel CSF1R variants. The *CSF1R* gene spans more than 60 kbp and contains 21 exons (transcript ID: ENST00000675795.1). It encodes a protein of 972 amino acids (protein ID: ENSP00000501699.1), containing five extracellular immunoglobulin domains, a transmembrane domain as well as an intracellular tyrosine kinase domain. The six novel variants are all located in the tyrosine kinase domain. Their respective amino acids are evolutionarily conserved, as confirmed through alignment with several species. The schematic displays the names and RefSeq IDs of the species used for the alignment with the human reference sequence of the CSF1R protein

The variants were annotated using wANNOVAR (wannovar.wglab.org) [28] and analyzed using multiple computational software programs to predict the effect of these variants (Functional Analysis Through Hidden Markov Models (FATHMM) [29], MutationTaster [30], Sorting Intolerant from Tolerant (SIFT) [31], Polymorphism Phenotyping v2, Human Variants Database (Polyphen2 HVAR) [32], Likelihood Ratio Test (LRT), Combined Annotation Dependent Depletion (CADD) [33], Mendelian Clinically Applicable Pathogenicity (M-CAP) [34]). Identified variants were classified following the recommendations of the American College of Medical Genetics and Genomics (ACMG) [35]. The current literature (up to June 2024) was searched using the NCBI National Library of Medicine (PubMed) to determine that the variants were not reported previously. Genetic data are summarized in Fig. 1 and Table 2.

**Table 2:** Genetic data and variant scoring

Patient ID	Patient 1	Patient 2	Patient 3	Patient 4	Patient 5	Patient 6	Patient 7
Mutation information							
Gene	*CSF1R*	*CSF1R*	*CSF1R*	*CSF1R*	*CSF1R*	*CSF1R*	*CSF1R*
Chromosome	5	5	5	5	5	5	5
Exon (NM_001288705.3)	17	15–21	12	16	18	12	19
Mutation site DNA (NM_001288705.3)	c.2384G>T	c.2133_2919del	c.1837G>A	c.2304C>A	c.2517G>T	c.1837G>A	c.2642C>T
Mutation site Protein (P07333-1)	p.Gly795Val	p.?	p.Val613Met	p.Phe768Leu	p.Trp839Cys	p.Val613Met	p.Ala881Val
Genomic start Gh37	149435840	149437155	149441075	149436865	149435626	149441075	149434812
Genomic start Gh38	150056277	150057592	150061512	150057302	150056063	150061512	150055249
Type of mutation	Missense	Deletion	Missense	Missense	Missense	Missense	Missense
Domain	Protein kinase domain	Protein kinase domain	Protein kinase domain | ATP binding site	Protein kinase domain	Protein kinase domain	Protein kinase domain | ATP binding site	Protein kinase domain
Variant scoring							
gnomAD_genome_ALL	Not listed	Not listed	Not listed	Not listed	Not listed	Not listed	Not listed
FATHMM_converted_rankscore	0.834	–	0.898	0.845	0.945	0.898	0.830
FATHMM_pred	D	–	D	D	D	D	D
MutationTaster_converted_rankscore	0.491	–	0.810	0.810	0.810	0.810	0.810
MutationTaster_pred	D	–	D	D	D	D	D
SIFT_converted_rankscore	0.274	–	0.912	0.912	0.784	0.912	0.721
SIFT_pred	T	–	D	D	D	D	D
Polyphen2_HVAR_rankscore	0.971	–	0.971	0.916	0.818	0.971	0.728
Polyphen2_HVAR_pred	D	–	D	D	D	D	D
LRT_converted_rankscore	0.353	–	0.445	0.629	0.504	0.445	0.372
LRT_pred	N	–	D	D	D	D	N
CADD_raw_rankscore	0.795	–	0.916	0.887	0.949	0.916	0.929
CADD_phred	27.1	–	32	29.7	34	32	33
M-CAP_rankscore	0.905	–	0.980	0.936	0.993	0.980	0.899
M-CAP_pred	D	–	D	D	D	D	D
ACMG criteria	PM1, PM2, PP3, PP4	PM1, PM2, PM4	PM1, PM2, PM5, PP3	PM1, PM2, PM6, PP3	PM1, PM2, PP3, PP4	PM1, PM2, PM5, PP3, PP4	PM1, PM2, PP3, PP4
ACMG classification	Likely pathogenic	Likely pathogenic	Likely pathogenic	Likely pathogenic	Likely pathogenic	Likely pathogenic	Likely pathogenic

FATHMM, Functional Analysis Through Hidden Markov Models, rankscore range 0 (neutral) – 1 (deleterious) [29]. MutationTaster, rankscore range 0 (neutral) – 1 (deleterious) [30]. SIFT, Sorting Intolerant From Tolerant, rankscore range 0 (tolerated) – 1 (deleterious) [31]. Polyphen2 HVAR, Polymorphism Phenotyping v2 (human variants database), rankscore range 0 (tolerated) – 1 (deleterious) [32]. LRT, Likelihood Ratio Test, rankscore range 0 (neutral) – 1 (deleterious). CADD, Combined Annotation Dependent Depletion, rankscore range 0 (potentially benign) – 1 (potentially pathogenic) [33], phred score range 0 (potentially benign) – 48 (potentially pathogenic) [33]. M-CAP, Mendelian Clinically Applicable Pathogenicity, rankscore range 0 (tolerated) – 1 (deleterious) [34]. ACMG, American College of Medical Genetics and Genomics [35]; D, deleterious; N, neutral; T, tolerated

### Protein alignment

To determine the evolutionary conservation of the CSF1R protein, the amino acid sequence was compared among different species (human, RefSeq NP_001275634.1; chimpanzee, RefSeq XP_016809515.1; rhesus macaque, RefSeq XP_001107711.4; cattle, RefSeq NP_001068871.2; dog, RefSeq XP_546306.2; cat, NP_001009231.1; sheep, RefSeq XP_027826197.2; rat, RefSeq NP_001025072.1; mouse, RefSeq NP_001032948.2; chicken, RefSeq NP_001308446.2; frog, RefSeq NP_001008181.1) using Jalview (jalview.org) [36] (Fig. 1).

## Case reports

### Patient 1

This patient presented at the age of 47 years with a 2-year history of motor, behavioral and sensory symptoms. After a fall she had developed pain in her right knee and gait difficulties. The gait disturbance worsened, and she was diagnosed with multiple sclerosis 1 year after symptom onset. As the symptoms worsened despite corticosteroid treatment, she became wheelchair-bound within the following year, forcing her to retire and move to a nursery home. Further symptoms included indifferent behavior and impaired impulse control with compulsive shopping and hoarding, requiring the appointment of a legal guardian. In addition, she reported sensory disturbances in her left hand, which were interpreted as carpal tunnel syndrome. The patient’s mother was reported to have had a stroke in her late sixties, her father had no known neurological disease. Her sister was reported to be healthy.

In the neurological examination two years after symptom onset, the patient exhibited spastic hypertonia of the lower limbs and a slight action tremor that was more evident on the right side. She showed severe gait apraxia with difficulties standing up and walking; she could only take single steps when being supported. The Babinski reflex, Gordon’s sign and Oppenheim sign were positive. She also exhibited emotionless behavior.

Cognitive testing using Montreal Cognitive Assessment (MoCA) [37] revealed cognitive deficits (23/30 points). Cerebral magnetic resonance imaging (MRI) showed generalized confluent white matter lesions, brain atrophy, and diffusion restrictions. Laboratory blood and cerebrospinal fluid (CSF) analyses for routine parameters and neurosyphilis serology were unremarkable. Plasma levels for arylsulfatase A, β-galactosidase, β-hexosaminidase A, total β-hexosaminidase A & B, β-galactocerebrosidase and β-glucocerebrosidase were normal.

Six months later, she required help in most activities of daily living such as cooking and personal hygiene. She also showed symptoms of depression despite antidepressant medication. Her gait disturbance had progressed and she was unable to stand or walk independently. She also showed slightly hypometric saccadic eye movements and an overshoot in the finger-to-finger test as well as moderate dysdiadochokinesia. Her rapidly progressive cognitive impairment over the course of six months became evident in her MoCA score, which was then 12/30 points.

**Fig. 2:**
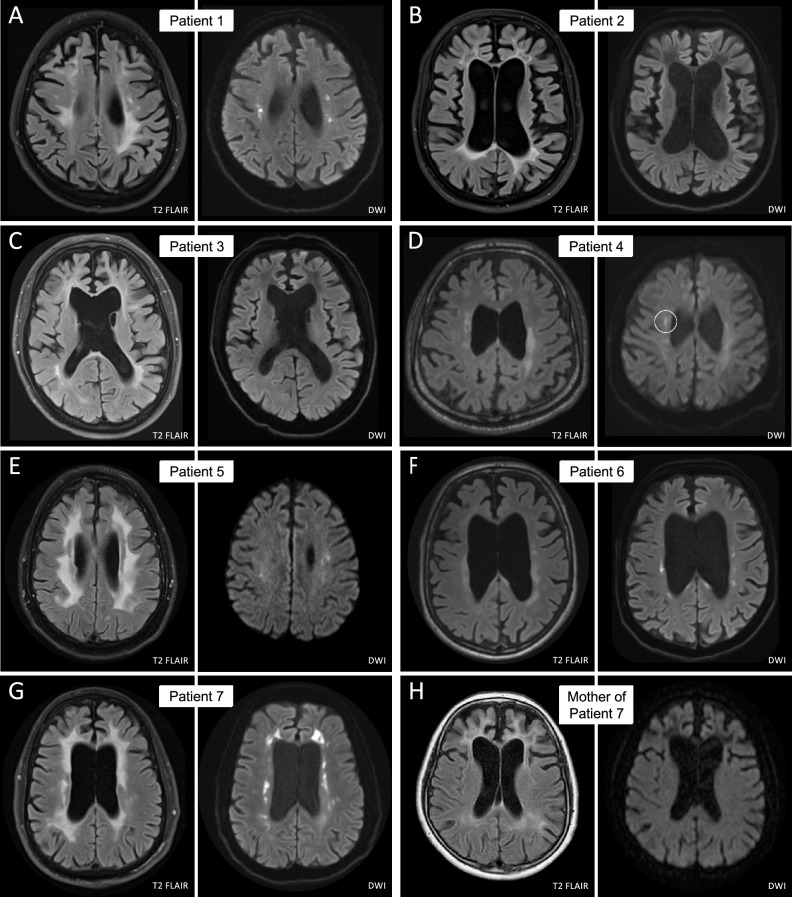
Cerebral magnetic resonance imaging. Magnetic resonance (MR) T2-weighted fluid-attenuated inversion recovery (FLAIR) as well as diffusion-weighted images (DWI) of every patient are depicted. The hallmark features of ALSP in MR imaging are patchy to confluent T2-hyperintensities mainly in the frontoparietal white matter while sparing the temporal lobe, supratentorial global brain atrophy accentuated in the frontal region as well as dispersed foci of restricted diffusion in the periventricular white matter on DWI sequences. All patients had white matter lesions and marked brain atrophy. In five of the patients, diffusion restriction was detected (Patients 1, 4 (circle), 5, 6, 7). All sites of diffusion restriction are hypointense in the apparent diffusion coefficient (ADC) sequence (data not shown)

The electrophysiological examination revealed a lesion of the pyramidal tract to the left arm and leg. The magnetic resonance imaging (MRI) of the brain showed supratentorial atrophy and white matter lesions as well as diffusion restriction (Fig. 2A). The neurodegenerative marker NfL was highly increased in serum and CSF (serum: 251 pg/mL, CSF: 19,081 pg/mL; controls [26]: serum: 24.3 ± 3.4 pg/mL, CSF 1052 ± 208.5 pg/mL).

Over the next 5 years, her condition deteriorated further; she was bedridden, unable to move, and mutistic at the age of 52 years.

Genetic testing revealed a missense variant in the *CSF1R* gene leading to an amino acid exchange in the CSF1R protein, c.2384G>T, p.Gly795Val (Fig. 1; Table 2).

DNA samples of the parents were not available; family history might suggest an affected mother, who was described to have had a stroke in her sixties. The variant of the patient was absent from the Genome Aggregation Database (gnomAD) [38] (PM2) and is located in the tyrosine kinase domain of the protein, which is known as a mutational hot spot (PM1). The amino acid glycine at this position (795) is conserved across different species [39] and is classified as deleterious by several in silico prediction programs (PP3). An amino acid exchange at one position before (794) has been described as likely pathogenic/pathogenic in the context of ALSP [40]. The clinical and MRI phenotype (diffusion restriction) is highly specific for ALSP (PP4). Taken together, the variant of the patient was classified as likely pathogenic according to ACMG criteria (PM1, PM2, PP3, and PP4).

### Patient 2

This patient presented at the age of 40 years with a 3-year history of progressive memory deficits, social withdrawal, indifference, aggressive behavior, poverty of speech, and incontinence for urine and stool. There was no family history of neurological disease. The patient had one sister and the parents were of Turkish origin.

In the clinical evaluation she showed orientation deficits, reserved behavior, executive dysfunction, apraxia and a slight dysdiadochokinesia on the left side. An MRI from the same year showed leukoencephalopathy, especially of the frontal lobe, atrophy of the corpus callosum as well as global atrophy. Infection serology, microbiologic, and virologic tests as well as CSF routine analysis and electrophysiology revealed no abnormalities. Plasma levels for arylsulfatase A, β-galactosidase, β-hexosaminidase A, total β-hexosaminidase A & B, β-galactocerebrosidase and β-glucocerebrosidase were normal.

One year later, at the age of 41 years, the patient needed help with most activities of daily living. She occasionally stumbled when climbing stairs. The patient sometimes left the house without notice and returned hours later. The neurological examination revealed a positive applause sign, marked poverty of speech, slight apraxia, dysdiadochokinesia and a slightly dysmetric heel–knee test. She had marked cognitive impairment (MoCA 8/30 points). NfL was highly elevated in serum and CSF (serum: 252 pg/mL, CSF: 14,786 pg/mL).

Over the course of the next 2 years, the gait worsened with parkinsonian features, apraxia became prominent, and cognitive impairment progressed (MoCA 3/30 points). The MRI of the brain showed white matter lesions and global atrophy, but no diffusion restriction (Fig. 2B).

Genetic testing revealed a heterozygous deletion of exon 15–21 in the *CSF1R* gene (Fig. 1; Table 2). The deletion affects a large part of the tyrosine kinase domain of the protein (PM1, PM4) and is absent from gnomAD (PM2). Taken together, the variant was classified as likely pathogenic according to ACMG criteria (PM1, PM2, and PM4).

### Patient 3

This patient experienced first symptoms at the age of 46 years, including poverty of speech, slowness in daily activities, and indifference. He also had difficulties executing his work as a forklift operator due to concentration deficits. The family history revealed neurologically healthy parents, but the patient’s maternal grandmother had suffered from early-onset dementia, categorized as arteriosclerotic, and had died at the age of 59 years.

When presenting at our institution 2 years later, the patient had no spontaneous speech production, and answered only to direct questions with “yes” or “no”. He needed help in many activities of daily living, such as shaving and showering, and suffered from a slight urinary incontinence. He was able to walk independently, although he was careful and slow when climbing stairs. In the neurological examination, he presented with pronounced apathy, apraxia, and slight action tremor of the right hand. The gait pattern was smooth, but the patient had a slightly stooped posture.

Cerebral MRI showed white matter lesions especially in the frontal lobe as well as global brain atrophy, most pronounced in the frontal lobe. Diffusion restriction was not detected (Fig. 2C). NfL was increased in serum and CSF (serum: 81 pg/mL, CSF: 6840 pg/mL). There were no other abnormalities in the routine CSF analysis and blood tests.

Genetic testing revealed a heterozygous missense variant in the *CSF1R* gene, c.1837G>A, p.Val613Met (Fig. 1; Table 2). The patient’s mother carried the same *CSF1R* variant but had not developed any symptoms at the time of examination (aged 68 years); however, the maternal grandmother had early-onset dementia (see above), suggesting an autosomal-dominant inheritance with incomplete penetrance in this family. The variant is located in the ATP binding site of the tyrosine kinase domain (PM1). It is not present in gnomAD (PM2). The affected amino acid is evolutionary highly conserved and classified as deleterious by several in silico prediction programs (PP3). Chu et al. described a variant in *CSF1R* at the same position, but with a different amino acid exchange (c.1837G>T, p.V613L) in the context of ALSP (PM5) [41]. Also, Berdowski et al. generated a zebrafish model carrying this variant, which exhibits pathological features found in ALSP brain tissue [42]. In addition, we found a second patient presenting with classical symptoms of ALSP and carrying the same variant (see Patient 6). The variant in our patient was classified as likely pathogenic according to ACMG criteria (PM1, PM2, PM5, and PP3).

### Patient 4

This patient had experienced first symptoms at the age of 40 years, including gait disturbances and impaired fine motor skills. Over the next 3 years, the symptoms had intensified. He was now wheelchair-bound and needed help with daily activities such as eating and body care. He had also developed swallowing difficulties and blurred speech. Further symptoms included depression, restlessness, anxiety, loss of appetite and weight, obstipation, and urinary urge. The family history war negative for neurological disorders.

In the clinical examination the patient showed marked signs of Parkinsonism with rigidity and strong freezing. He was not able to sit or stand up independently and was unable to walk due to freezing. In addition, reduced facial expression, dysarthria, apraxia, and dysdiadochokinesis were observed. He had marked anxiety. MoCA testing revealed cognitive impairment (17/30 points).

The cerebral MRI showed global atrophy, moderate white matter lesions, especially in the parietal lobe, and one site of diffusion restriction (Fig. 2D). Routine CSF analysis and blood tests were normal. NfL in serum and CSF was strongly elevated (serum: 42 pg/mL, CSF: 7363 pg/mL).

Genetic testing revealed a heterozygous missense variant in the *CSF1R* gene, c.2304C>A, p.Phe768Leu (Fig. 1; Table 2). Segregation analysis uncovered the absence of this variant in the parent’s genome, i.e., we assume the de novo occurrence of the variant in our patient (PM6). The variant is located in the tyrosine kinase domain of the protein (PM1) and is not listed in gnomAD (PM2). The amino acid at the corresponding location is highly conserved and the variant was rated as deleterious by several in silico prediction programs (PP3). The variant of the patient was classified as likely pathogenic according to ACMG criteria (PM1, PM2, PM6, and PP3).

### Patient 5

The patient started to have orientation difficulties when driving at the age of 46 years. His cognitive abilities rapidly deteriorated and he had to resign from his work as truck driver. His condition further worsened and he was transferred to a nursing home at the age of 48 years, where we first met him 1 year later. By then, aged 49 years, he had no spontaneous speech production and only communicated with nodding or “no”. He also suffered from epileptic seizures. Family history revealed that the patient’s mother suffered from slight memory deficits from the age of 75 years.

Clinical examination was limited due to the severity of his status. He had slight left-sided facial palsy. He was able to move his arms and legs spontaneously, but was unable to walk, even with assistance, most likely due to apraxia. He had no prominent swallowing difficulties. In the following 2 years, he lost his residual ability to communicate as well as his ability to sit and was confined to bed. He developed severe dysphagia. He died shortly after his 51st birthday.

Cerebral MRI at the age of 46 and 47 years showed bilateral white matter hyperintensities on FLAIR sequence, multiple areas of diffusion restriction in subcortical and central white matter on DWI as well as global brain and corpus callosum atrophy (Fig. 2E). NfL in serum and CSF was strongly elevated (serum: 352 pg/mL, CSF: 14,563 pg/mL).

Genetic testing uncovered a heterozygous missense variant in the *CSF1R* gene, c.2517G>T, p.Trp839Cys (Fig. 1; Table 2). Segregation analysis revealed the same variant in the mother, who was clinically unaffected at the time of genetic testing. She developed slight memory deficits at the age of 75 years. The variant is located in the tyrosine kinase domain of the protein (PM1) and is not listed in gnomAD (PM2). The amino acid at the corresponding location is highly conserved and the variant was rated as deleterious by several in silico prediction programs (PP3). MRI showed diffusion restriction which together with the clinical course is very specific for ALSP (PP4). The variant of the patient was classified as likely pathogenic according to ACMG criteria (PM1, PM2, PP3, and PP4).

### Patient 6

The patient first noticed a limited walking distance at the age of 51 years and pain in her legs after exertion. The gait difficulties worsened and she additionally developed fine motor impairment. At her first visit in our outpatient clinic, aged 54 years, the patient was only able to walk with a walker. She reported being clumsy and having an unclear handwriting; she also suffered from progressive dysarthria for six months. She had urge symptoms and infrequent urge incontinence for urine. She was still able to perform all activities of daily living. Family history revealed that the patient’s mother had developed similar symptoms at around 50 years of age including dysarthria and gait disturbance. She died at the age of 56 years.

Clinical examination of our patient revealed saccadic eye movements and hypermetric saccades. She had a mixed dysarthria with pseudobulbar and cerebellar components. Motor examination showed mild weakness in arm and hip abduction, and pyramidal signs (mild spasticity, increased reflexes, bilaterally positive Babinski sign). She had mild dysmetria with discrete intention tremor, bradydysdiadochokinesia as well as bradykinesia and decrement in finger tapping. Her walking was broad-based with small steps and reduced arm swinging.

When the patient presented again 1 year later, she was no longer able to walk; she had been permanently dependent on a wheelchair since the age of 55 years. The fine motor impairment, particularly of the left hand, had worsened further, and she needed assistance with eating. Due to her progressive dysarthria, the patient no longer spoke on the phone. Her husband reported pathological laughter and mood swings with increased verbal aggression. The patient and her husband had not noticed any memory deficits. Previously, she had undergone treatment with up to 600 mg of L-Dopa for 1 month, but it proved ineffective.

During clinical evaluation, the patient mostly communicated in 1–2-word sentences. The clinical examination was limited due to apraxia, motor initiation difficulties, and moderate aphasia. She had frontal release signs (applause sign, palmomental reflex, pathological laughter). Dysarthria was mixed, with occasional words difficult to understand. She showed slight involuntary movements and an increased muscle tone in her left side. She had pyramidal signs (brisk reflexes with clonic responses, Babinski’s sign on the left). The finger-nose test revealed a slight intention tremor on the right side and was apractic on the left side. Fingertapping was irregular on the right side, while it was not possible on the left side due to apraxia/motor initiation impairment. The patient was unable to independently transfer from wheelchair to bed. She was only able to stand and walk with significant support from two people due to a combination of gait-induced spasticity, leg apraxia, extrapyramidal-motor disorder, and significant retropulsion. Cognitive performance assed by MoCA was impaired (23/30 points).

Cerebral MRI showed an advanced neurodegenerative process with significant atrophy of gray and white matter, including the corpus callosum, as well as diffusion restriction (Fig. 2F). Except for protein (72 mg/dl), routine CSF parameters were unremarkable, but NfL was highly increased (9756 pg/ml).

Genetic testing revealed a heterozygous missense variant in the *CSF1R* gene, c.1837G>A, p.Val613Met (Fig. 1; Table 2). This is the same variant we found in Patient 3 (PM1, PM2, PM5, and PP3). MRI showed diffusion restriction which together with the clinical course and the family history is very specific for ALSP (PP4). The variant was classified as likely pathogenic according to ACMG criteria (PM1, PM2, PM5, PP3, and PP4).

### Patient 7

The patient had first symptoms at the age of 44 years with a change in personality including physical aggression. In addition, he was hospitalized twice due to depression. Shortly thereafter, he lost his job as electrician since he could no longer meet the demands of the work. He further developed a significant lack of drive, requiring him to move back in with his parents. He also showed signs of an impulse control disorder with uncontrolled purchases on the internet. Activities of daily living could still be performed independently. Family history revealed that his mother had no apparent motor deficits, but slight memory difficulties at the time of presentation, aged 68 years, that later progressed to severe dementia, requiring her translocation to a nursing home. The maternal grandfather of the patient died at the age of 53 years; the death was attributed to long-term consequences of his service at war. The patient’s maternal grandmother, originally from Slovenia, died at the age of 61 years due to heart disease.

When presenting the first time at our hospital at the age of 46 years, neurological examination revealed no physical deficits but moderate apraxia as well as cognitive deficits (MoCA 15/30 points).

Cerebral MRI showed white matter lesions in the frontal and parietal lobe, global atrophy as well as prominent diffusion restriction (Fig. 2G). NfL in CSF was highly increased (7043 pg/ml).

Genetic testing uncovered a heterozygous variant c.2642C>T, p.Ala881Val in the *CSF1R* gene (Fig. 1; Table 2). The patient’s mother carried the same variant. Her cerebral MRI revealed extensive confluent hyperintensities in the superior frontal gyri and global brain atrophy accentuated in the frontal region; no diffusion restriction was detected (Fig. 2H). The variant was absent from gnomAD (PM2) and is located in the tyrosine kinase domain of the protein, which is known as a mutational hot spot (PM1). The amino acid alanine at this position (881) is conserved across different species [39] and is classified as deleterious by several in silico prediction programs (PP3). The clinical and MRI phenotype (diffusion restriction) is highly specific for ALSP (PP4). Taken together, the variant was classified as likely pathogenic according to ACMG criteria (PM1, PM2, PP3, and PP4).

## Discussion

Since the identification of *CSF1R* as the causal gene for ALSP, over 100 pathogenic/likely pathogenic variants have been reported in association with the disorder [7, 9]. Comprehensive understanding of the range of pathogenic variants and the diversity of symptoms they elicit is essential in rare genetic diseases such as ALSP. To this end, we report seven patients carrying six hitherto undescribed *CSF1R* variants associated with ALSP, and thereby contribute to the expanding spectrum of ALSP genetics and phenomenology.

Five of the six novel variants reported here are missense variants located either in the ATP-binding site or the tyrosine kinase domain of the receptor (Fig. 1). Given that these variants occur in evolutionarily conserved positions, they are considered critical for maintaining proper receptor function, i.e., the autophosphorylation of the intracellular domain. This is further substantiated by the predictions made by the applied rating tools (FATHMM, MutationTaster, SIFT, Polyphen2, LRT, CADD, M-CAP), most of which indicated that all of the missense variants were deleterious (Table 2). Although missense mutations in the kinase domain of *CSF1R* are presumed to cause loss-of-function [43–45], their heterozygous presence is sufficient to manifest the disease. This indicates a dominant-negative mode of action, possibly due to the receptor's obligate dimeric configuration. However, the pathomechanism of the missense mutations remains a topic of ongoing debate [42, 44, 46–48]. In one patient (Patient 2), a heterozygous deletion spanning exon 15–21 was identified. This large deletion has the potential to cause mRNA decay or generate a truncated protein. Theoretically, mRNA decay would lead to haploinsufficiency, whereas the production of a truncated protein would likely cause a dominant-negative effect since the deletion includes the kinase domain. Notably, Patient 2 exhibits a comparatively slow progression of the disease; this could be indicative of mRNA decay with resultant haploinsufficiency as a possible scenario in this patient. However, a comprehensive analysis of molecular genetics in conjunction with clinical phenotype and disease progression in larger cohorts is required to establish a correlation between genotype and phenotype in *CSF1R*-related ALSP.

The family histories of the patients described here reflect the heterogeneity of inheritance and penetrance of *CSF1R* mutations in ALSP. In two families (Patients 2 and 4), family history was informative and negative. This raises the possibility of a de novo* CSF1R* mutation in these patients. De novo mutations in ALSP are a known phenomenon and account for up to 30% of ALSP cases [8, 10]. In one of these families (Patient 4), segregation analysis confirmed the de novo occurrence of the *CSF1R* variant by excluding the variant in both parents. Genetic testing was performed in three other families (Patient 3, Patient 5, and Patient 7). The results showed that the three mothers were carriers of the *CSF1R* variant. Two of them, the mothers of Patient 3 and Patient 5, were both in their late 60 s and asymptomatic at the time of testing. The third, mother of Patient 7, had slight memory difficulties aged 68 years, and later developed severe dementia. Her MRI exhibited classical features of ALSP with confluent white matter lesions and frontally accentuated brain atrophy (Fig. 2H). Clinical presentation with progressive cognitive impairment and MRI features is compatible with findings in ALSP, even though onset would be comparatively late and progression slow. The mother of Patient 5 developed mild memory and walking deficits at the age of 75 years. While in these two persons, especially in the mother of Patient 7, cognitive decline could indicate the manifestation of ALSP, it is also possible that the impairment resulted from another form of dementia. As the average age at onset for ALSP in women is 40 years [9, 10], and there is a known age-dependent penetrance with 95% of the carriers diseased by the age of 60 years [10], a manifestation beyond the age of 65 years seems unlikely, but is not impossible. In one of the families with the mother as asymptomatic carrier (Patient 3), the maternal grandmother was likely affected by ALSP, as she suffered from early-onset dementia and died at the age of 59 years, suggesting an incomplete penetrance in the patient’s asymptomatic mother. Only in one family (Patient 6), even though not confirmed genetically, there was a clear pattern of dominant inheritance with the mother showing typical ALSP symptoms at an early age. In conclusion, the study’s findings on the differences in family history, inheritance, and penetrance, highlight the intricate nature of ALSP genetics, emphasizing the importance of performing *CSF1R* genetic testing, even in cases where the family history is negative or suggests a recessive inheritance. Moreover, the high percentage of asymptomatic, oligosymptomatic, or late-onset carriers in this cohort is intriguing and demands further investigation into the protective factors that take effect in these individuals.

From a clinical perspective, the hallmark features of ALSP consist of cognitive impairment and motor dysfunction [9, 10]. Among the seven patients examined in this study, four displayed cognitive impairment as the first sign of the disease, while the remaining three exhibited gait difficulties as their initial symptom (Table 1). With the exception of one patient (Patient 6), who was only examined in the early stage of her disease course, all other patients are known to have progressed to severe dementia. All patients developed motor impairment. The combination of cognitive and motor impairment lead to a significant nursing effort and to wheelchair dependence, or even confinement to bed. Additionally, this study frequently observed symptoms congruent with cases reported in the literature, including apraxia as well as pyramidal and extrapyramidal signs such as spasticity and parkinsonism [9, 10, 49–53]. As the disease progressed, the patients frequently suffered from dysarthria, dysphagia, incontinence, and, in one case, seizures, which is also in line with previously described cases of ALSP [9, 10, 50, 51, 54]. Interestingly, one patient exhibited pronounced anxiety, and another showed pathological laughing, both of which have not yet been described as typical symptoms in ALSP.

Cerebral MRI scans of all seven patients showed frontoparietal white matter lesions and brain atrophy, which are typical features of ALSP [9, 10, 45, 53, 55]. Additionally, diffusion restriction was detected in five of the patients, which is another characteristic finding of the disease [9, 10, 21]. Besides the typical clinical signs and characteristic features in MR imaging, the determination of NfL in serum and/or CSF constitutes the third pillar in the diagnostic workup of ALSP. While NfL serves as a general neuronal biomarker found elevated in various neurological conditions [56], it is exceptionally increased in ALSP [26]. The CSF NfL levels of the patients included in this study ranged from 6840 to 19,081 pg/mL, which significantly exceeds the upper standard value of approximately 1000 pg/mL [26], reflecting the rapid neuronal loss in these patients.

In summary, our study expands the genetic spectrum of ALSP by identifying six previously undescribed variants in seven patients displaying characteristic symptoms of the disease. The observed variability in genetic background and penetrance across the studied families underscores the crucial role of genetic testing, even in cases with no prior family history. Since rare diseases are frequently caused by genetic alterations, genetic testing is often the only way to establish a definitive diagnosis, highlighting its importance in the diagnostic process. Therefore, we emphasize the significance of early inclusion of genetic testing in the diagnostic workup of rare diseases and especially leukoencephalopathies of undefined origin.

## Data Availability

Data will be shared upon qualified request.
